# Retraction: The analysis and solution for intercity travel behaviors during holidays in the post-epidemic era based on big data

**DOI:** 10.1371/journal.pone.0330561

**Published:** 2025-08-25

**Authors:** The PLOS One Editors

The *PLOS One* Editors retract this article [1] because it was identified as one of a series of submissions for which we have concerns about potential manipulation of the publication process, peer review integrity, and authorship. These concerns call into question the validity and provenance of the reported results. We regret that the issues were not identified prior to the article’s publication.

All authors either did not respond directly or could not be reached.

## References

[1] Zhang X, Gao J. The analysis and solution for intercity travel behaviors during holidays in the post-epidemic era based on big data. PLoS One. 2023;18(7):e0288510. doi: 10.1371/journal.pone.0288510 37467244 PMC10355389

